# Immune‐related gene signature predicts clinical outcomes and immunotherapy response in acute myeloid leukemia

**DOI:** 10.1002/cam4.4687

**Published:** 2022-03-30

**Authors:** Qiang Xu, Dedong Cao, Bin Fang, Siqi Yan, Yu Hu, Tao Guo

**Affiliations:** ^1^ Institute of Hematology, Union Hospital, Tongji Medical College Huazhong University of Science and Technology Wuhan China; ^2^ Collaborative Innovation Center of Hematology Huazhong University of Science and Technology Wuhan China; ^3^ Department of Oncology Renmin Hospital of Wuhan University Wuhan China; ^4^ Department of Nephrology, Union Hospital, Tongji Medical College Huazhong University of Science and Technology Wuhan China

**Keywords:** acute myeloid leukemia, biomarker, immune microenvironment, immunotherapy, prognosis

## Abstract

**Background:**

The immune response in the bone marrow microenvironment has implications for progression and prognosis in acute myeloid leukemia (AML). However, few immune‐related biomarkers for AML prognosis and immunotherapy response have been identified. We aimed to establish a predictive gene signature and to explore the determinants of prognosis in AML.

**Methods:**

Immune‐related genes with clinical significance were screened by a weighted gene co‐expression network analysis. Seven immune‐related genes were used to establish a gene signature by a multivariate Cox regression analysis. Based on the signature, low‐ and high‐risk groups were compared with respect to the immune microenvironment, immune checkpoints, pathway activities, and mutation frequencies. The tumor immune dysfunction and exclusion (TIDE) method was used to predict the response to immune checkpoint blockade (ICB) therapy. The Connectivity Map database was used to explore small‐molecule drugs expected to treat high‐risk populations.

**Results:**

A seven‐gene prognostic signature was used to classify patients into high‐ and low‐risk groups. Prognosis was poorer for patients in the former than in the latter. The high‐risk group displayed higher levels of immune checkpoint molecules (LAG3, PD‐1, CTLA4, PD‐L2, and PD‐L1), immune cell infiltration (dendritic cells, T helper 1, and gamma delta T), and somatic mutations (*NPM1* and *RUNX1*). Moreover, hematopoietic stem cell/leukemia stem cell pathways were enriched in the high‐risk phenotype. Compared with that in the low‐risk group, the lower TIDE score for the high‐risk group implied that this group is more likely to benefit from ICB therapy. Finally, some drugs (FLT3 inhibitors and BCL inhibitors) targeting the expression profiles associated with the high‐risk group were generated using Connectivity Map.

**Conclusion:**

The newly developed immune‐related gene signature is an effective biomarker for predicting prognosis in AML and provides a basis, from an immunological perspective, for the development of comprehensive therapeutic strategies.

## Graphical abstract

The progression of bioinformatics tools has accelerated the search for biomarkers associated with acute myeloid leukemia (AML) prognosis. This article aims to originate an immune‐associated risk signature for prognostic analysis of AML and explore the mutation characteristics, immune characteristics, and immunotherapy response defined by risk signature.

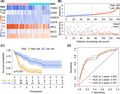

## 1 INTRODUCTION

Acute myeloid leukemia (AML) is the most commonly occurring aggressive leukemia in adults that is characterized by uncontrolled proliferation of immature myeloid cells and diverse clinical features.^1^ Although most patients with AML initially respond to chemotherapy, half of all patients relapse within 5 years of diagnosis.^2^ Recurrence after standard induction chemotherapy is a key obstacle to the treatment of AML; furthermore, some patients do not respond to induction therapy.^3^ Given the increasing incidence of AML and the low survival rate, better prognostic biomarkers are needed for the development of prevention, screening, and treatment approaches.^4^ Recently, researchers have identified numerous prognostic markers on public databases.^5^, ^6^ These studies also proved that a comprehensive prognostic analysis of multiple features is more valuable than an individual feature.

There is increasing evidence that various immune pathways are activated in AML, leading to immunosuppressive effects, altering the tumor immune microenvironment, and reducing overall survival (OS) rates.^7^, ^8^ The immunosuppressive tumor microenvironment is known to significantly impede the anti‐leukemia immune responses. Moreover, this immunosuppression also adversely affects and invalidates the regular and experimental treatments.^9^ An in‐depth understanding of basic immunity and immune escape mechanisms in the periphery and bone marrow microenvironment can accelerate the identification of biomarkers that can predict clinical outcomes.^10^ Immunotherapy induces a specific immune response to inhibit and kill tumor cells, thereby reducing the rate of tumor recurrence.^11^ Comprehensive studies of immunophenotypes in the AML microenvironment may improve our understanding of anti‐tumor responses and provide a basis for clinically effective immunotherapies.^12^ However, few patients benefit from immunotherapy and there are only a few effective markers to accurately predict the patient's response to immunotherapy.^13^ Therefore, this study is intended to perform a comprehensive analysis of immune‐related genes associated with AML, to propose novel potential biomarkers for the clinical prognosis and ICB therapy responsiveness of AML.

In this study, we used the weighted gene co‐expression network analysis to first categorize the immune‐related genes associated with the clinical characteristics of AML. We then assessed the merit of these genes for the efficient prediction of clinical outcomes in patients with AML. We analyzed AML transcriptomic data from multiple patient cohorts to develop an immune‐related signature for the prediction of prognosis and response to immunotherapy. Gene expression‐based immune cell quantification was performed, and the relationships between immune cell subtypes and risk level based on the established signature were assessed. Overall, these data indicate that the identified signature might be a practical indicator for predicting heterogeneous clinical behavior and prognosis in AML.

## 2 MATERIALS AND METHODS

### 2.1 Data acquisition and preprocessing

Expression profiles and clinical information for 151 AML samples were downloaded from The Cancer Genome Atlas (TCGA) database (https://tcga‐data.nci.nih.gov/tcga/). The following additional datasets were used for validation: GSE 37642 (*n* = 417) and GSE 146173 (*n* = 246) from the Gene Expression Omnibus (GEO) database (https://www.ncbi.nlm.nih.gov/geo/). A list of immune‐related genes (IRGs) was derived from the gene set “IMMUNE SYSTEM PROCESS” in the Molecular Signatures Database (MSigDB, https://www.gsea‐msigdb.org/gsea/msigdb/) and ImmPort (http://www.immport.org/). For the TCGA dataset, only highly variable IRGs with a median absolute deviation (MAD) higher than 1.0 were selected for further analyses.

### 2.2 Weighted gene co‐expression network analysis

The R package “WGCNA” was used to construct co‐expression modules relevant to prognosis in the TCGA AML dataset. After filtering out samples with significant deviations in expression values using the hclust function, a hierarchical clustering analysis was performed to cluster the retained samples based on clinical information. The pickSoftThreshold function was used to estimate the optimal soft threshold power parameter (β) when constructing a scale‐free network and then an adjacency matrix was generated. Next, a dissimilarity matrix (1‐TOM) based on the topological overlap measure (TOM) was derived from the adjacency matrix. IRGs with similar expression patterns were classified into the same module using 1‐TOM as a proximity metric, and co‐expression modules were identified using the cutreeDynamic function. Each module contained at least 35 genes, and modules with high similarity were merged with a cut height of 0.25. Different modules were represented by different colors. The correlations between clinical traits and module eigengenes (MEs) were evaluated using a Pearson correlation analysis. Finally, the most representative module correlated with AML prognosis was selected for further analyses.

### 2.3 Construction and validation of a prognostic risk signature

Univariate Cox proportional hazards regression and Kaplan–Meier analyses were used to screen the eligible IRGs in the turquoise module. Additionally, only genes present in the three datasets were considered for the construction of a prognostic risk signature. To select the most representative IRGs, a least absolute shrinkage and selection operator (LASSO) Cox regression analysis was performed. The “ComBat” function was used to correct data from TCGA and GEO expression profiles to eliminate potential batch effects between platforms. TCGA AML samples were chosen as the training cohort. A multivariate Cox regression analysis was performed to derive the regression coefficients for the IRGs. The risk score for each sample was calculated using the expression level and regression coefficient of the finally determined IRGs to develop a risk signature for prognostic prediction. Each sample was assigned a risk score, and samples were classified into a high‐risk group and a low‐risk group, using the median risk score of the training cohort as the threshold for stratification. A scatter plot and risk score distribution plot were generated to describe sample characteristics and a heatmap was used to visualize gene expression levels. The difference in OS between the high‐risk and low‐risk groups was identified by a Kaplan–Meier analysis. The accuracy of the risk signature for OS prediction was determined using the receiver operating characteristic (ROC) curve. In addition, TCGA AML cases were grouped according to clinical features, including age (<60 years and ≥60 years), sex (male and female), cytogenetic risk (poor, favorable, and intermediate), and *FLT3*, *DNMT3A*, *NPM1*, *TP53*, and *RUNX1* mutation statuses to further evaluate prognostic differences between risk score subtypes. AML samples were assigned to a risk group (favorable, intermediate, or poor) according to the level of clinical risk determined by cytogenetic abnormalities.^2^ GSE 37642 (*n* = 417) and GSE 146173 (*n* = 246) were included as two validation cohorts to verify the risk signature's performance. In the TCGA and GSE 146173 cohorts, clinical features (age, cytogenetic risk, and gene mutations) were included in a multivariate analysis to determine whether the risk score was an independent predictor.

### 2.4 Estimation of the immune microenvironment

To explore the possible reasons for the differential prognosis of the risk subgroups (low‐ and high‐risk groups) distinguished by the seven‐IRG signature, the immune microenvironment of the high‐risk group and the low‐risk group was analyzed. Based on the ESTIMATE algorithm implemented in the R package, immune scores and ESTIMATE scores were extracted from the patient expression matrix from TCGA. Differences in scores and immune checkpoint expression between the high‐risk and low‐risk groups were evaluated. An immune cell abundance identifier (ImmuCellAI, http://bioinfo.life.hust.edu.cn/ImmuCellAI/), a method based on a gene expression matrix, was used to accurately estimate the abundance of 24 immune cell subtypes.^14^ The Wilcoxon test was used to compare the levels of infiltration of immune cell subtypes between risk subgroups, and the results were visualized using the “fmsb” R package. The tumor immune dysfunction and exclusion (TIDE) algorithm was used. Patients with higher TIDE scores are more likely to show immune escape; therefore, TIDE scores were compared between the risk subgroups to predict the response to immune checkpoint (PD‐1, CTLA4) blockade therapy. Detailed methodological information can be found in previous reports.^15^

### 2.5 Clinical characteristics and gene set variation analysis

To further explain the prognostic differences of risk subgroups, the mutation statuses of genes with high mutation frequencies in AML were evaluated.^16^ The chi‐square test was used to compare the distribution differences of clinical information and gene mutation status between risk subgroups in the TCGA cohort. GSVA was used to evaluate variation in pathway activities in an unsupervised manner.^17^ Using the “limma” package in R, the *t*‐test was used to compare pathway scores between high‐risk and low‐risk groups, and only the subgroup‐specific pathways with adjusted *p*‐value <0.05 are presented in the Results.

### 2.6 Prognostic analysis of gene expression profiles

A Kaplan–Meier survival analysis was used to characterize differences in survival with respect to IRG expression levels. To explore the connections between the expression of IRGs and clinical characteristics, the Kruskal–Wallis test was used. Immune scores from the tumor microenvironment were obtained and GSVA was used to derive gene set activity scores. The associations between gene expression levels and the aforementioned scores were tested by Spearman's correlation analysis, and data were visualized using the “ggplot2” package.

### 2.7 Connectivity Map analysis

The connectivity map (CMap) database was used to identify drugs expected to reverse the input differential expression profile.^18^ To explore potential treatments for high‐risk populations, we analyzed differentially expressed genes (DEGs) between high‐ and low‐risk groups and used DEGs to explore small‐molecule drugs based on CMap (https://clue.io). DEGs were obtained by setting |log fold change (FC)| > 1 and false discovery rate adjusted *p*‐value <0.05 as thresholds. The DEGs were obtained using the “limma” package in R.

### 2.8 Statistical analysis

Survival curves were compared using the Kaplan–Meier method. The Wilcoxon test was used to compare continuous variables between the two groups. The correlations between gene expression and clinical characteristics were tested using the Kruskal–Wallis test. Differences in the distributions of clinical characteristics and the status of highly mutated genes between risk subgroups were identified using the chi‐square test. Relationships between parameters were evaluated using Spearman's correlation test. A *p*‐value of less than 0.05, was considered to be significant unless stated otherwise. All data were analyzed using R (V.4.0.0).

## 3 RESULTS

### 3.1 Module construction and screening

**FIGURE 1:**
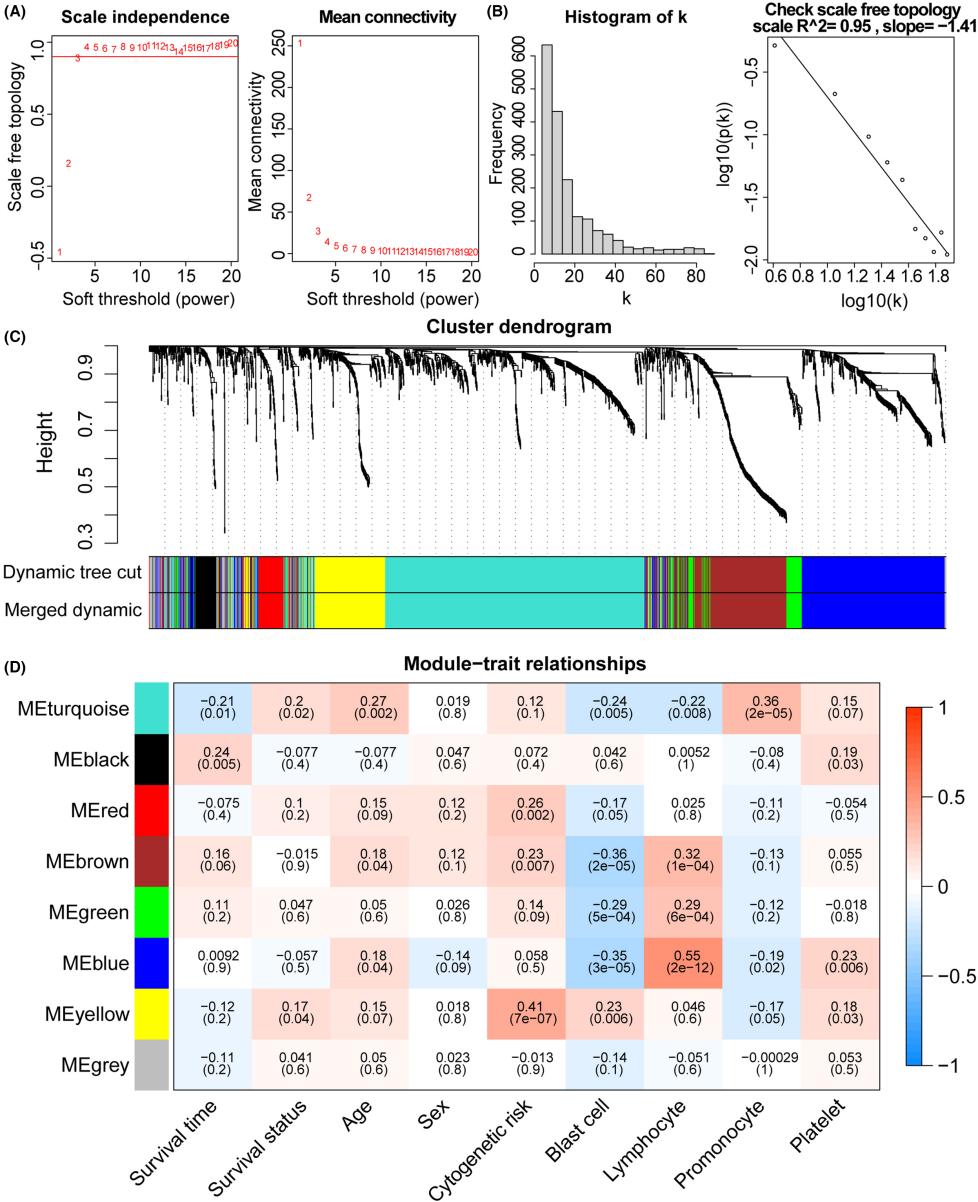
Co‐expression module recognition. (A) Scale independence and mean connectivity under different threshold conditions. (B) Scale‐free topology (*β* = 4). (C) A cluster dendrogram of co‐expressed genes was obtained by average linkage hierarchical clustering based on 1–TOM. (D) Module‐trait relationship plot. The numbers in each grid are Pearson correlation coefficients and *p*‐values. Positive correlations are shown in red, while negative correlations are shown in blue. TOM: topological overlap measure

To avoid IRGs with low variation across samples, we retained genes with an MAD exceeding 1.0, resulting in a total of 1813 IRGs. Nine TCGA samples were excluded owing to a lack of survival time data. Using the hclust function, TCGA‐AB‐2987 was identified as an outlier and was excluded (Figure S1A). All matching samples were clustered by average linkage and Pearson correlation distances (Figure S1B). We used a soft threshold power of 4 to build a scale‐free topology (scale‐free *R* ^2^ = 0.95) (Figure 1A,B). For the combination of modules, setting a cut height of 0.25, we proved that no modules showed a high similarity (Figure 1C). Subsequently, we obtained seven co‐expression modules from the cluster tree, with 57 to 662 IRGs (Figure 1D). The associations between module eigengenes and clinical traits were defined using the Pearson correlation coefficient (PCC). The module showing the highest correlations with survival time (PCC = −0.21, *p* = 0.01) and survival status (PCC = 0.2, *p* = 0.02) was the turquoise module (*n* = 662; Table S1). Therefore, IRGs in the turquoise module were included in further in‐depth analyses.

### 3.2 Screening of prognostic immune‐related genes

Among 662 genes in the turquoise module, 427 genes shared between the TCGA and GEO datasets were screened. By univariate Cox proportional hazard regression and Kaplan–Meier analyses, 11 IRGs were included in further analyses (Table S2), using *p*‐value cutoffs of 0.01 and 0.05. A LASSO Cox regression analysis was performed, where seven IRGs *(CALR*, *PSMD3*, *THBS1*, *BST2*, *MPO*, *OGFR*, and *CDK6*) were used to construct the risk signature (Figure S2).

### 3.3 Construction of a risk signature in the TCGA cohort

**FIGURE 2:**
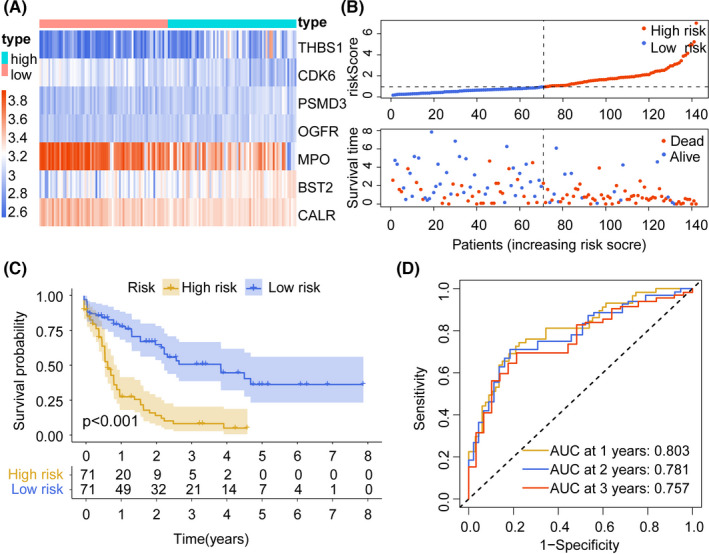
Construction of risk signature based on the TCGA cohort. (A) Gene expression in TCGA risk subgroups. (B) Comparison of various characteristics between the two groups of patients in the TCGA dataset. (C) Kaplan–Meier survival analysis (*p* < 0.001) results for 142 patients in the TCGA dataset. (D) ROC curve based on the risk score in the TCGA dataset

By a multivariate Cox regression analysis, an immune‐related risk signature was established to predict the prognosis of patients with AML. The risk score was calculated using the following formula based on seven IRGs: Risk score = BST2*0.184823 + MPO*(−0.139860) + PSMD3*1.018944 + THBS1*0.031434 + CALR*(−0.250446) + OGFR*(−0.765625) + CDK6*(−0.592075). The median risk score was used as a threshold to divide the training cohort into a high‐risk group (*n* = 71) and a low‐risk group (*n* = 71). We visualized the risk scores and gene expression profiles of the risk subgroups (Figure 2A,B). The Kaplan–Meier curve indicated that the survival rate was lower in the high‐risk group than in the low‐risk group (*p* < 0.001; Figure 2C). The areas under the ROC curve (AUCs) of the risk score for predicting 1‐, 2‐, and 3‐year OS were 0.803, 0.781, and 0.757, respectively, suggesting that the risk score has an excellent predictive value (Figure 2D).

### 3.4 Strong prognostic prediction ability of the risk signature

**FIGURE 3:**
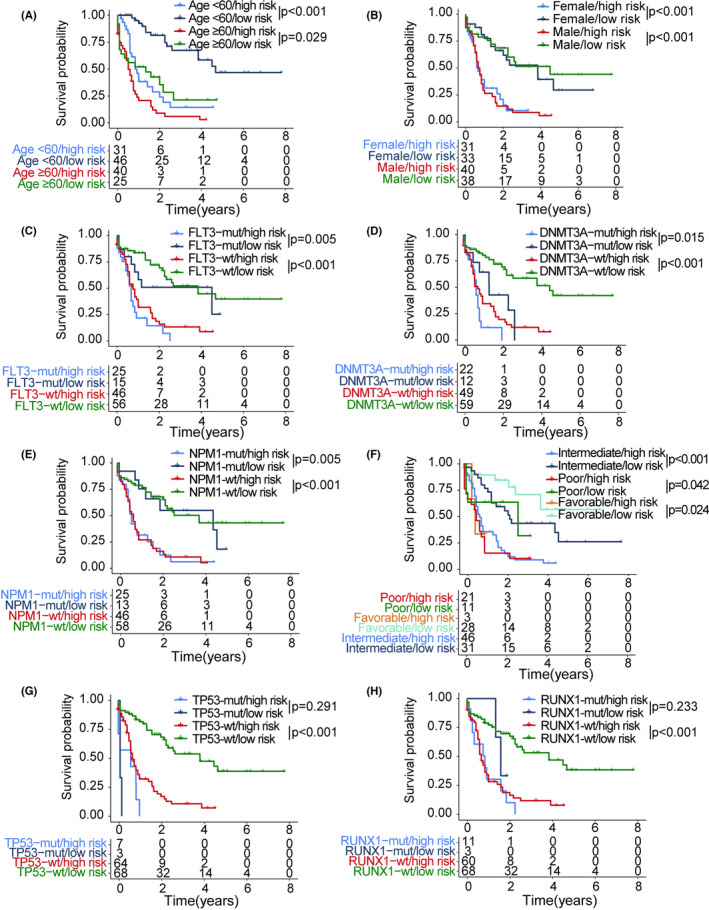
Stratified survival analysis of risk score subtypes in clinical subgroups. (A–H) Samples were grouped according to age (<60 years and ≥60 years), sex (male and female), FLT3 status, DNMT3A status, NPM1 status, cytogenetic risk (poor, favorable, and intermediate), TP53 status, and RUNX1 status

**FIGURE 4:**
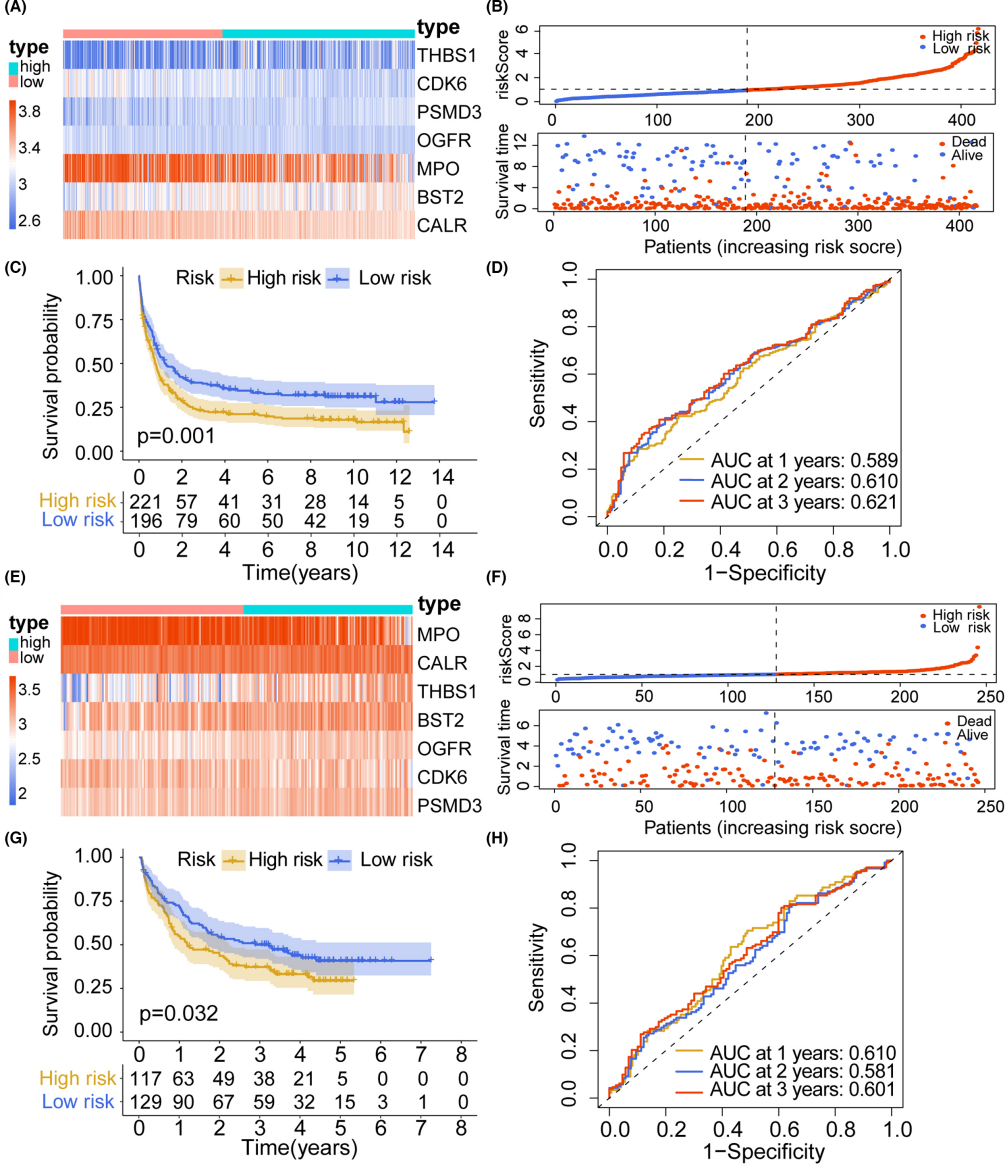
Validation of the risk signature in GEO cohorts. Trends in gene expression, risk score, and survival status in the risk subgroups in the GSE 37642 (A, B) and GSE 146173 (E, F) cohorts. Kaplan–Meier survival analysis of risk score subtypes in the GSE 37642 (C) and GSE 146173 (G) cohorts. The area under ROC curve based on risk scores in the GSE 37642 (D) and GSE 146173 (H) cohorts

In a stratified survival analysis of OS in different risk groups and TCGA clinical subgroups, age (≥60 years & high risk vs. ≥60 years & low risk, *p* = 0.029; <60 years & high risk vs. <60 years & low risk, *p* < 0.001), sex (female & high risk vs. female & low risk, *p* < 0.001; male & high risk vs. male & low risk, *p* < 0.001), *FLT3* status (*FLT3* mutant & high risk vs. *FLT3* mutant & low risk, *p* = 0.005; *FLT3* wildtype & high risk vs. *FLT3* wildtype & low risk, *p* < 0.001), *DNMT3A* status (*DNMT3A* mutant & high risk vs. *DNMT3A* mutant & low risk, *p* = 0.015; *DNMT3A* wildtype & high risk vs. *DNMT3A* wildtype & low risk, *p* < 0.001), *NPM1* status (*NPM1* mutant & high risk vs. *NPM1* mutant & low risk, *p* = 0.005; *NPM1* wildtype & high risk vs. *NPM1* wildtype & low risk, *p* < 0.001), cytogenetic risk (poor & high risk vs. poor & low risk, *p* = 0.042; intermediate & high risk vs. intermediate & low risk, *p* < 0.001; favorable & high risk vs. favorable & low risk, *p* = 0.024), *TP53* wild‐type status (*TP53* mutant & high risk va. *TP53* mutant & low risk, *p* = 0.291; *TP53* wildtype & high risk vs. *TP53* wildtype & low risk, *p* < 0.001), and *RUNX1* wild‐type status (*RUNX1* mutant & high risk vs. *RUNX1* mutant & low risk, *p* = 0.233; *RUNX1* wildtype & high risk vs. *RUNX1* wildtype & low risk, *p* < 0.001) did not affect the performance of risk score in OS prediction (Figure 3A–H). Due to the small sample size of the *TP53* mutation group (*n* = 10) and the *RUNX1* mutation group (*n* = 14), we found no differences in prognosis between risk score subtypes in these groups (Figure 3G,H). The grouping criterion for validation cohorts was based on the median risk score of the training cohort. For the GSE 37642 dataset, we visualized the risk score and gene expression characteristics of the high‐risk group (*n* = 221) and the low‐risk group (*n* = 196) (Figure 4A,B). Similarly, low survival rates in the high‐risk groups were also observed in this validation cohort (*p* = 0.001; Figure 4C). The AUCs of the risk score for predicting 1‐, 2‐, and 3‐year OS were 0.589, 0.610, and 0.621, respectively, supporting the predictive value of the risk score (Figure 4D). Using the GSE 146173 dataset, we visualized the risk score and gene expression characteristics of the high‐risk and low‐risk groups (Figure 4E,F). An increase in the risk score was associated with a poor prognosis. The high‐risk group (*n* = 117) had a worse prognosis than that of the low‐risk group (*n* = 129) (*p* = 0.032; Figure 4G). The AUCs of the risk score for predicting 1‐, 2‐, and 3‐year OS were 0.610, 0.581, and 0.601, respectively, supporting the predictive value of the risk score (Figure 4H). Multivariate analysis of TCGA (*p* < 0.001) and GSE 146173 (*p* = 0.046) cohorts showed that the risk score was still associated with OS when adjusted for age, cytogenetic risk, *NPM1* status*, DNMT3A* status, *FLT3‐*ITD, *FLT3‐*TKD, *TP53* status, *RUNX1* status, *CEBPA* status, *IDH1* status, *IDH2* status, and *ASXL1* status (Table 1).

**TABLE 1:** Univariate analysis and multivariate analysis of the correlation of risk score with overall survival

Parameters	Univariate analysis			Multivariate analysis		
	HR	95% CI	*p*	HR	95% CI	*p*
TCGA set						
Age (<60/≥60)	3.11	2.02–4.77	<0.001	2.87	1.77–4.65	<0.001
Cyto risk(poor/favorable + intermediate)	1.87	1.15–3.03	0.012	1.31	0.74–2.32	0.349
NPM1 mutation (yes/no)	0.77	0.49–1.22	0.261			
DNMT3A mutation (yes/no)	0.46	0.29–0.75	0.002	0.68	0.38–1.20	0.182
FLT3‐ITD (yes/no)	1.36	0.81–2.26	0.243			
FLT3‐TKD (yes/no)	2.19	1.18–4.06	0.013	2.63	1.20–5.76	0.016
TP53 mutation (yes/no)	4.71	2.37–9.35	<0.001	3.63	1.59–8.28	0.002
RUNX1 mutation (yes/no)	1.84	0.99–3.41	0.052			
CEBPA mutation (yes/no)	1.12	0.54–2.31	0.767			
IDH1 mutation (yes/no)	0.81	0.39–1.68	0.570			
IDH2 mutation (yes/no)	1.01	0.52–1.96	0.967			
ASXL1 mutation (yes/no)	1.71	0.42–6.99	0.452			
Risk score (high/low)	3.64	2.31–5.72	<0.001	2.96	1.75–5.00	<0.001
GEO set (GSE 146173)						
Age (<60/≥60)	2.26	1.63–3.13	<0.001	2.05	1.44–2.90	<0.001
Cyto risk(poor/favorable + intermediate)	2.21	1.51–3.22	<0.001	1.73	1.05–2.85	0.031
NPM1 mutation (yes/no)	0.54	0.38–0.78	0.001	0.70	0.44–1.11	0.127
DNMT3A mutation (yes/no)	1.22	0.87–1.71	0.248			
FLT3‐ITD (yes/no)	0.93	0.62–1.41	0.742			
FLT3‐TKD (yes/no)	0.70	0.39–1.27	0.244			
TP53 mutation (yes/no)	3.32	2.02–5.46	<0.001	2.30	1.21–4.37	0.011
RUNX1 mutation (yes/no)	1.87	1.29–2.71	0.001	1.52	0.94–2.45	0.089
CEBPA mutation (yes/no)	0.45	0.14–1.42	0.173			
IDH1 mutation (yes/no)	1.43	0.84–2.43	0.191			
IDH2 mutation (yes/no)	1.38	0.88–2.18	0.159			
ASXL1 mutation (yes/no)	1.52	0.99–2.34	0.056			
Risk score (high/low)	1.29	1.11–1.50	0.001	1.19	1.00–1.40	0.046

Abbreviations: CI, Confidence interval; Cyto risk, cytogenetic risk; HR, hazard ratio.

### 3.5 Immune landscape between high‐ and low‐risk AML groups

**FIGURE 5:**
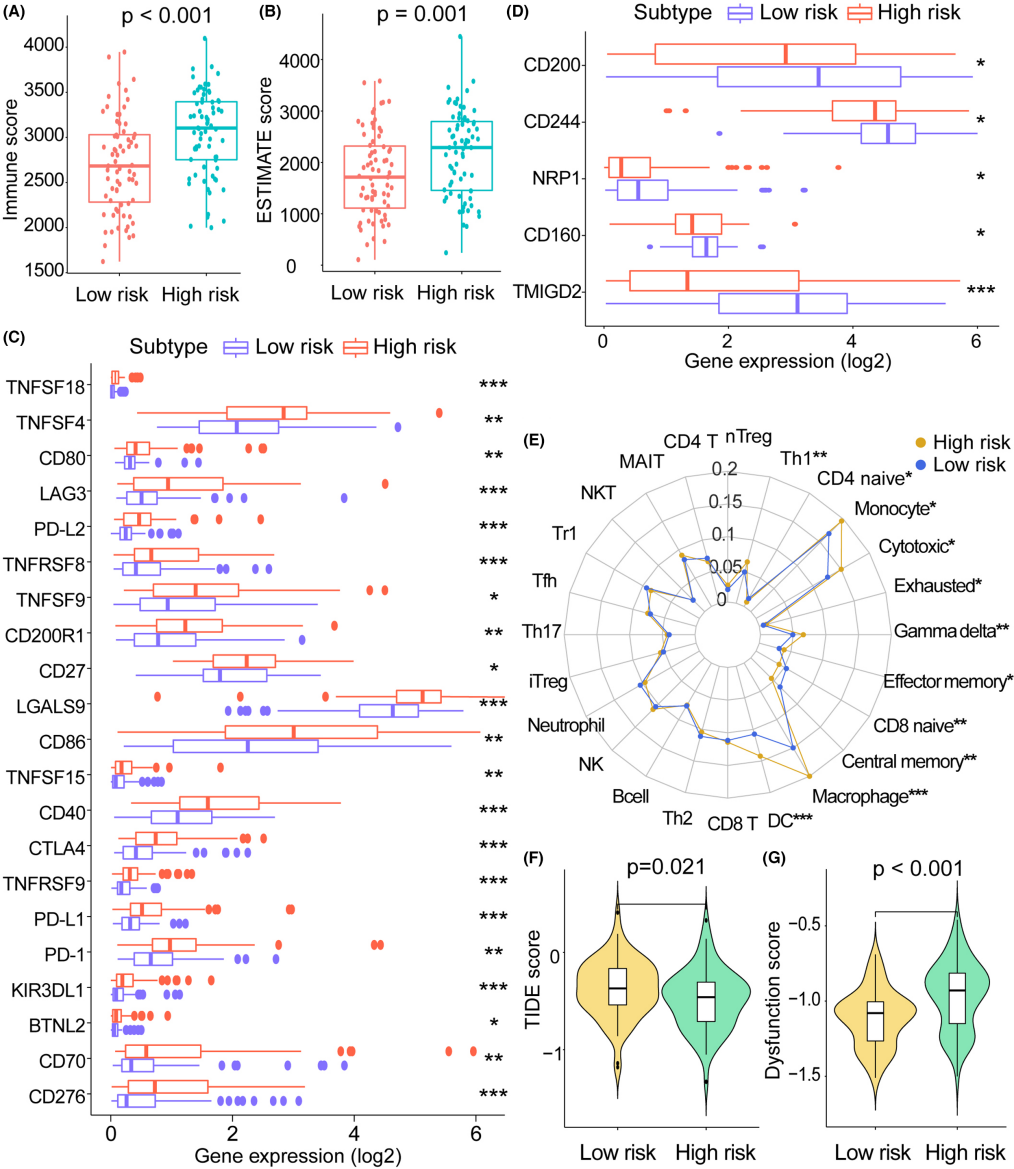
Landscapes related to immunity in TCGA risk subgroups. (A) Immune scores in high‐ and low‐risk groups derived from the ESTIMATE algorithm. (B) ESTIMATE scores in high‐ and low‐risk groups. (C, D) Differences in the expression levels of immune checkpoints between risk subgroups. Immune checkpoints with similar expression trends were classified into a single group. (E) Abundances and differences of 24 immune cells in risk subgroups. (F) Differences in TIDE scores between high‐ and low‐risk groups. (G) Differences in T cell dysfunction scores between high‐ and low‐risk groups. TIDE, tumor immune dysfunction and exclusion. *p < 0.05, **p < 0.01, ***p < 0.001

Immune cells in the tumor microenvironment have been implicated in tumor progression, immunotherapy, and patient outcomes. We assessed differences in the tumor microenvironment between high‐risk and low‐risk groups. The high‐risk AML group had higher immune scores (*p* < 0.001; Figure 5A) and ESTIMATE scores than those of the low‐risk group (*p* = 0.001; Figure 5B). We characterized differences in the expression levels of immune checkpoint genes between the risk subgroups. Figure 5C illustrates the immune checkpoint genes with higher expression in high‐risk AML than in low‐risk AML, and Figure 5D illustrates the immune checkpoints with high expression in low‐risk AML. Using ImmuCellAI, we obtained the immune cell abundance in each sample to compare immune cell infiltration levels between risk subgroups (Figure 5E). Nine immune cell subtypes, including T helper 1, CD4 naïve T cells, monocytes, cytotoxic T cells, exhausted T cells, gamma delta T cells, effector memory T cells, macrophages, and dendritic cells, showed higher abundances in the high‐risk group than in the low‐risk group. In addition, CD8 naïve T cells and central memory T cells showed higher levels of infiltration in the low‐risk group than in the high‐risk group. The TIDE score (*p* = 0.021) was higher in the low‐risk group, whereas the T‐cell dysfunction score (*p* < 0.001) was higher in the high‐risk group (Figure 5F,G).

### 3.6 Correlation between TCGA AML subgroups and somatic mutations

**FIGURE 6:**
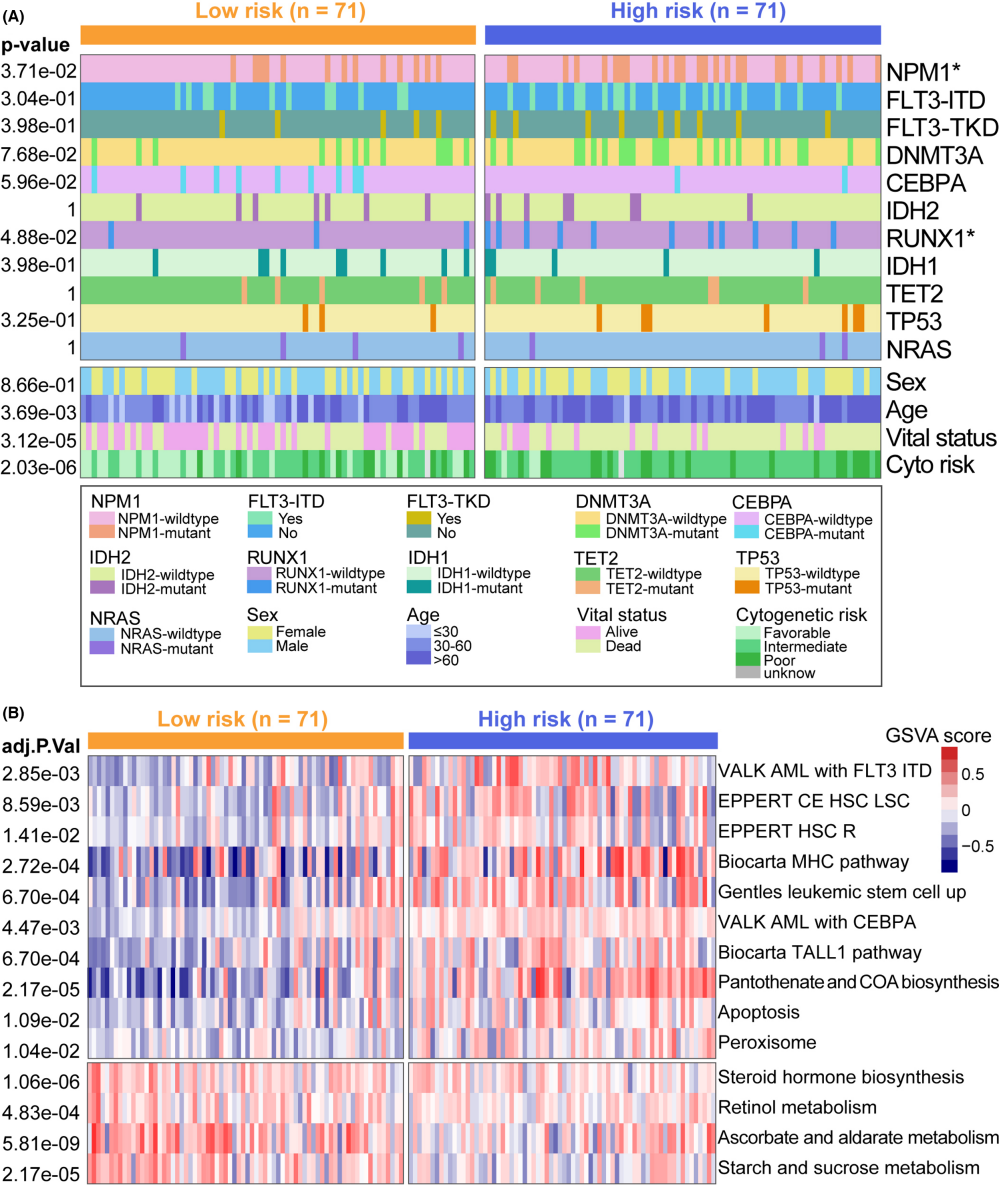
Heatmap of patient characteristics and GSVA. (A) The distributions of mutation frequencies and clinical characteristics in high‐risk and low‐risk groups of TCGA samples. (B) GSVA of the two subgroups. GSVA, gene set variation analysis

We analyzed the potential reasons for the prognosis differences of risk subgroups, and the distribution of the top‐ranked mutated genes and clinical features between subgroups were visualized using a heatmap (Figure 6A). Chi‐square tests revealed several significant differences between AML subgroups. The mutation frequencies of *NPM1* (*p* = 3.71e‐02), *RUNX1* (*p* = 4.88e‐02), *DNMT3A* (*p* = 7.68e‐02), and *FLT3‐*ITD (*p* = 3.04e‐01) were higher in the high‐risk group than in the low‐risk group, although the differences in *FLT3‐*ITD and *DNMT3A* mutation frequencies were not statistically significant. No significant differences in the mutation statuses of *CEBPA* (*p* = 5.96e‐02), *IDH2* (*p* = 1), *IDH1* (*p* = 3.98e‐01), *TET2* (*p* = 1), *TP53* (*p* = 3.25e‐01), and *NRAS* (*p* = 1) were observed between the risk subgroups. Advanced age (*p* = 3.69e‐03), death status (*p* = 3.12e‐05), and worse cytogenetic risk (*p* = 2.03e‐06) were correlated with the high‐risk group. Sex (*p* = 8.66e‐01) was not correlated with the risk subtypes.

### 3.7 Differential pathways in patients with AML

Based on the risk status, we implemented a GSVA of the subgroups to obtain enriched biological pathways in the two subgroups. Hematopoietic stem cells/Leukemia stem cells, AML with *CEBPA* mutation, AML with *FLT3*‐internal tandem duplication mutation, TALL1 pathway, and MHC pathway, apoptosis were enriched in the high‐risk group (Figure 6B). Ascorbate and aldarate metabolism, retinol metabolism, and starch and sucrose metabolism were enriched in the low‐risk group (Figure 6B).

### 3.8 Clinical relevance analysis of genes

**FIGURE 7:**
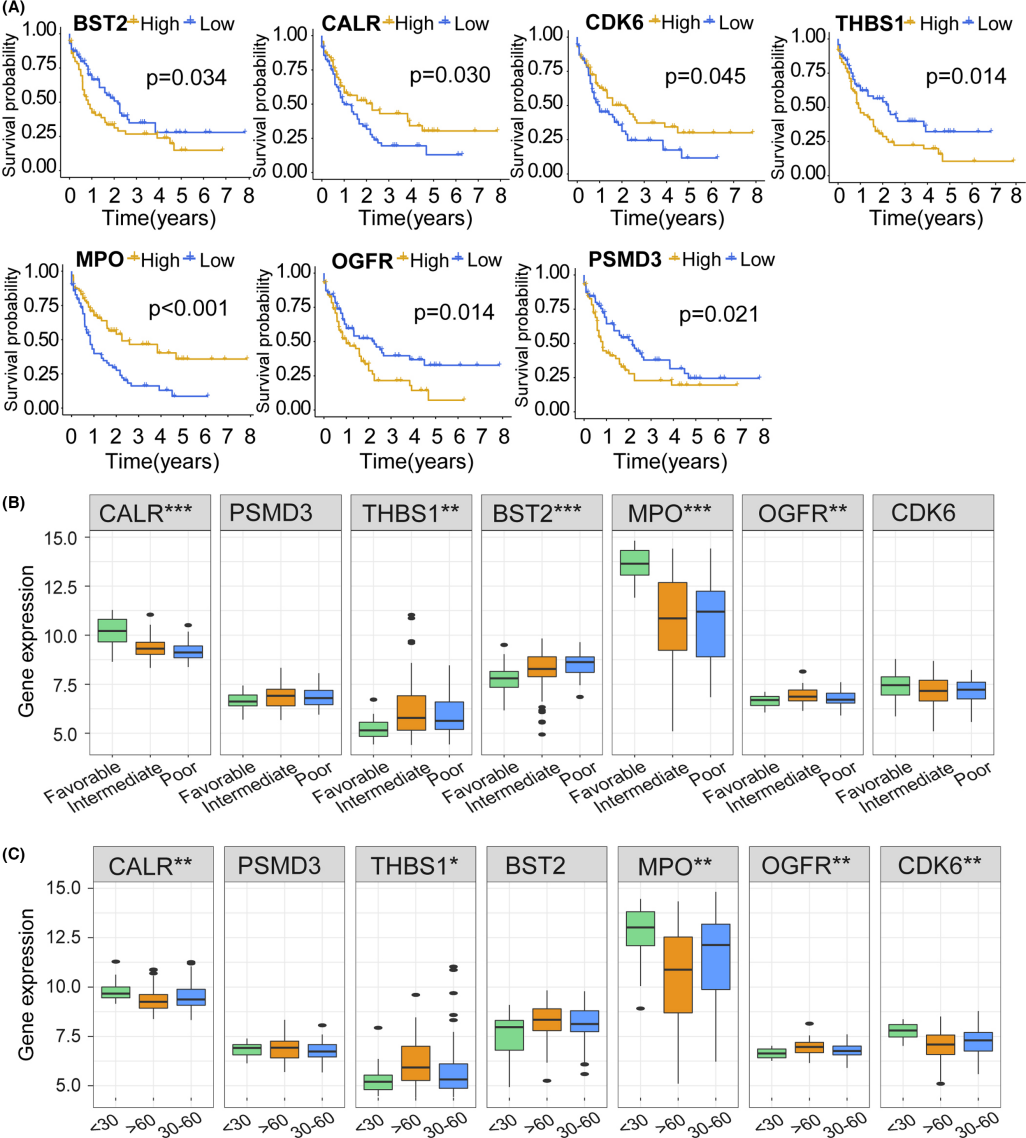
Clinical value of immune‐related genes. (A) Kaplan–Meier survival curves for genes. All samples were divided into a high expression group and a low expression group according to the median gene expression value. (B, C) Differences in the expression levels of genes with respect to cytogenetic risk and age. *p < 0.05, **p < 0.01, ***p < 0.001

As described earlier, a Kaplan–Meier survival analysis indicated that elevated expression levels of *BST2* (*p* = 0.034), *OGFR* (*p* = 0.014), *PSMD3* (*p* = 0.021), and *THBS1* (*p* = 0.014) were associated with a poor prognosis in AML, and low expression levels of *MPO* (*p* < 0.001), *CALR* (*p* = 0.030), and *CDK6* (*p* = 0.045) were associated with a poor OS (Figure 7A). Stratified by cytogenetic status, *CALR*, *THBS1*, *BST2*, *MPO*, and *OGFR* levels differed among three subgroups (poor cytogenetic groups, favorable cytogenetic groups, and intermediate cytogenetic groups) (Figure 7B). The differences in IRG expression among the three subgroups are shown in Figure 7C, according to age group (<30 years, 30–60 years, and >60 years). Finally, based on the GSVA scores for the gene set and immune scores, we evaluated correlations between IRG expression and scores (Figure S3).

### 3.9 Drug prediction based on CMap

**FIGURE 8:**
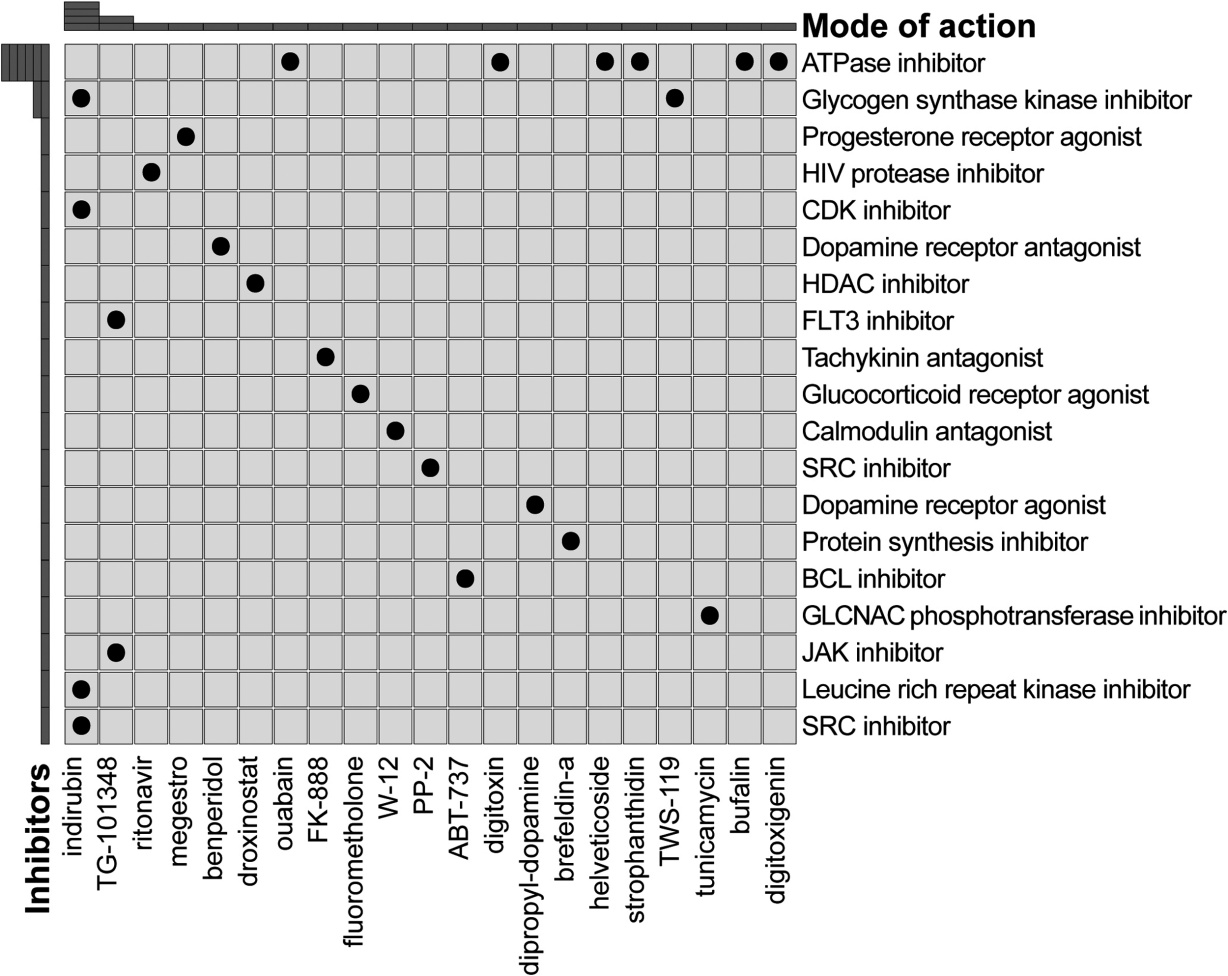
Connectivity Map (CMap) analysis. Row names indicated the mode of action, and column names corresponded to small‐molecule drugs

To identify compounds predicted to alter the characteristic gene expression profiles for high‐risk populations, the CMap database was used for the prediction of small‐molecule drugs based on the 50 most significantly upregulated DEGs and 50 most significantly downregulated DEGs in the high‐risk group. Finally, we screened 21 potential small‐molecule drugs and derived 19 mechanisms based on a mode‐of‐action (MoA) analysis (Figure 8). In particular, ABT‐737 acted as a BCL inhibitor, TG‐101348 functioned as a FLT3 inhibitor, and six drugs (digitoxigenin, bufalin, strophanthidin, digitoxin, ouabain, and helveticoside) acted as ATPase inhibitors. These compounds were derived from the IRG signature and are potential therapeutics for high‐risk AML.

## 4 DISCUSSION

Although cytogenetic risk stratification has been proposed in recent years, AML is a heterogeneous disease, especially in terms of the immune microenvironment, which leads to wide variation in prognosis.^19^, ^20^ Other clinical features such as age, performance status, and gene mutations (such as *NPM1*, *FLT3*, *DNMT3A*, *CEBPA*, *TP53*) are considered to be significant prognostic factors in patients with AML at initial diagnosis.^16^ To determine the AML subtypes associated with immune‐related genes and a better prognosis, we used a WCGNA to identify immune genes related to prognosis and established a prognostic risk signature based on seven genes associated with OS (*CALR*, *PSMD3, THBS1, BST2, MPO*, *OGFR,* and *CDK6*).

We verified the prognostic value of the IRG signature. When the median risk score was used to divide samples into two groups, the low‐risk group showed a significantly better OS based on the Kaplan–Meier survival curve. Stratified survival analysis revealed that the medium risk score can still determine high‐risk and low‐risk patients in different clinical subgroups. More importantly, based on the TCGA cohort and GSE 146173 dataset, the risk score was still significantly associated with the OS in AML, even after adjustment for clinical factors (age, cytogenetic risk, and gene mutations). These results suggest that the risk signature we constructed has good application value in prognosis prediction. After that, we sought to parse out clinical features of patients with high‐risk scores, the high‐risk group displayed a strong tendency toward advanced age, poor cytogenetic risk, and *RUNX1* mutation, all of which were indicative of a poor prognosis in AML.^16^, ^21^ We also noticed that the high‐risk group showed a higher frequency of *FLT3* mutations, although the difference was not statistically significant. *FLT3* mutations include internal tandem duplications (ITDs) and tyrosine kinase domain (TKD). However, the predictive value of these mutations for prognosis remains controversial.^21^

Calreticulin (*CALR*) is a major Ca^2+^‐binding protein in the endoplasmic reticulum. *CALR* mutations may inhibit the anti‐tumor effect of ICB by inhibiting the phagocytic function of dendritic cells.^22^ Thrombospondin 1 (*THBS1*) encodes an adhesive glycoprotein that mediates cell‐to‐cell interactions and is necessary for efficient CD47 activation, which induces the overexpression of pro‐inflammatory osteopontin in early monocyte‐derived macrophages.^23^ Bone marrow stromal cell antigen 2 (*BST2*) is involved in the growth and development of B cells. Type I interferons upregulate *BST‐2*, thus reducing natural killer (NK) cell responses to HIV‐1‐infected cells.^24^ Myeloperoxidase (*MPO*) is a heme protein synthesized during myeloid differentiation; it constitutes the major component of neutrophil azurophilic granules and is a fundamental component of the innate immune response against microbial pathogens.^25^ The OGF‐OGFR axis can regulate the degree of CD3^+^ T‐cell infiltration in the central nervous system.^26^ Cyclin‐dependent kinase 6 (*CDK6*) can promote the expression of pro‐inflammatory factors (IL‐17 and IL‐36) by phosphorylating EZH2.^27^ In addition, *CDK6*, as an effector of TCR, drives proliferation in Treg cells.^28^ In summary, the IRG signature was associated with the immune response.

Subsequently, we explored the relationships between the risk subgroups, tumor immune microenvironment, and immune checkpoint genes. We observed that the high‐risk group had a higher immune score and a higher ESTIMATE score than those of the low‐risk group, suggesting that immune cells are more abundant in the high‐risk group. The risk subgroups defined by the median risk score had different immune checkpoint expression patterns, with higher frequencies of *PD‐1*, *PD‐L1*, *PD‐L2*, *CTLA‐4*, and *LAG3* expression in high‐risk populations. Patients with high levels of immune checkpoints are more likely to develop T‐cell exhaustion, leading to an immunosuppressive microenvironment and a worse prognosis.^12^, ^29^ In addition, interferon gamma derived from NK cells upregulates MHC I, leading to resistance to the anti‐cancer efficacy of NK cells.^30^ The increase in exhausted T cells and the decline in central memory T cells may be indicators of recurrence after hematopoietic stem cell transplantation (HSCT) in the high‐risk group.^31^ Therefore, the high‐risk group with high immune checkpoint expression and exhausted T‐cell infiltration exhibited a poor prognosis, consistent with our results. In the tumor microenvironment, the binding of programmed cell death 1 (PD‐1) and PD‐L1 has a negative‐modulating effect on T cells and reduces the production of cytokines, thereby inhibiting cytotoxic T‐cell‐mediated anti‐tumor immunity and tumor clearance ability.^32^, ^33^ Unfortunately, high *PD‐1* expression often results in an exhausted T‐cell phenotype, leading to immune escape and poor outcomes.^29^ The immunosuppressive mechanism and effect of cytotoxic T lymphocyte antigen 4 (*CTLA4*) also show resemblance with *PD‐1*.^34^ ICB therapy is still a very promising method to cure AML; in particular, immune genes provide a basis for the identification of candidate targets for AML ICB therapy.^35^ The expression of immune checkpoints has been considered an indicator of prognosis in patients receiving immunotherapy.^36^, ^37^ The roles of *PD‐1* and *PD‐L1* in immunosuppression in cancers make them potential targets for ICB therapy.^38^

In a recent phase 2 study, ICB therapy (nivolumab) concurrent with azacytidine resulted in a higher objective response rate, longer median OS, and longer event‐free survival than those of chemotherapy alone.^39^ In addition, PD‐L1 can increase the efficacy of other treatments when used in combination with them.^40^, ^41^ In our study, the high‐risk group had lower TIDE scores and higher T cell dysfunction scores; accordingly, the greater response to ICB therapy might be explained by higher expression levels of immune checkpoints.

To gain a comprehensive view of the immunological nature of AML subtypes, we obtained somatic mutations in both groups. *NPM1* mutation frequency was higher in the high‐risk group than in the low‐risk group and might be an immunotherapy target.^42^ We found that the number of elderly patients differed significantly between the risk subgroups, and more precisely, with more elderly patients in the high‐risk group.^43^ The T cells of patients with AML showed signs of aging and exhaustion at the time of diagnosis.

Aging involves shortened telomere ends; however, other factors can induce telomerase‐independent senescence.^44^, ^45^ Leukemia stem cells are defined by their role in the initiation of leukemia and their unique immune resistance characteristics.^46^ Treatment with a PARP1 inhibitor and NK cell transfer can inhibit leukemia in mouse models.^47^ We found that leukemia stem cell‐related pathways are enriched in the high‐risk group.

A CMap analysis accurately identified targeted inhibitors known to have specific effects on AML, including a BCL inhibitor (ABT‐737),^48^ FLT3 inhibitor (TG‐101348),^49^ ATPase inhibitor,^50^ HDAC inhibitor (droxinostat),^51^ and CDK inhibitor (indirubin).^52^ These compounds are candidates for the treatment of high‐risk AML. Given that the survival rate of patients treated with a single immune checkpoint inhibitor or targeted therapy is not ideal, the combination of the two may produce long‐term effects.

Our study has many highlights. Several researches previously have proposed certain risk signatures based on various characteristics, in hopes of stratifying the prognosis of cancer patients.^53^, ^54^ Zheng et al. have identified a signature of seven‐lncRNA to predict the OS of patients with AML.^6^ Compared with those studies, the WGCNA used in our study has a unique advantage in dealing with gene expression data because it allows us to gain insight into the connection between coexpression modules and clinical characteristics of the disease. Moreover, our risk signature can distinguish between low‐risk and high‐risk patients in different clinical subgroups, regardless of their age, sex, cytogenetic risk, *NPM1*, *DNMT3A*, and *FLT3* mutation status. Alrisk signature can be used to predict the responsiveness of patients with AML to ICB therapy. ICB therapy is a breakthrough in cancer treatment, but its clinical benefit is restricted to a limited range of patients. Therefore, based on our risk signature, we predict a potential therapeutic strategy with drugs that target the gene expressions associated with high‐risk populations. This may reveal potential features for developing a comprehensive treatment regime for AML patients in near future.

Although our study provides insight into the impact of immune responses in AML, it had several limitations. First, the predictive risk signature was generated based on data obtained from TCGA and GEO, and complete information for all potentially relevant parameters could not be obtained for each patient. Second, clinical information and expression profile data were obtained from different sources, and differences in analysis processes may have affected the accuracy of the study results. Third, our research results were derived from the analysis of public data, and these results were not validated at the cellular and molecular levels using in vitro/in vivo experiments with active patient samples.

## 5 CONCLUSION

In summary, we identified a promising immune‐related prognostic biomarker for AML. The newly established AML subtypes and biomarkers are potential predictive indicators for the response to ICB therapy; however, in‐depth studies are needed to validate these findings.

## CONFLICT OF INTEREST

The authors report no conflict of interest.

## AUTHOR CONTRIBUTION

QX completed the diagrams, interpreted the results, and wrote the manuscript. DC and BF provided the suggestions. SY analyzed the data. YH and TG designed the study and revised the manuscript. All authors read and approved the final manuscript.

## ETHICS STATEMENT

Not applicable.

## Supporting information


Figure S1
Figure S2
Figure S3

Table S1
Table S2

## Data Availability

The data used to support the findings of this study are downloaded from The Cancer Genome Atlas database (https://portal.gdc.cancer.gov/), Gene Expression Omnibus (https://www.ncbi.nlm.nih.gov/geo/), ImmPort (http://www.immport.org/), and Molecular Signatures Database (https://www.gsea‐msigdb.org/gsea/msigdb/). The data are available from the corresponding authors on reasonable request.
