# Supplementary material for: Immune‐related gene signature predicts clinical outcomes and immunotherapy response in acute myeloid leukemia
Source: Cancer Med. 2022 Mar 30;11(17):3364–80. doi: 10.1002/cam4.4687 (PMC9468431; doi:10.1002/cam4.4687)
Supplement: Supplementary file 1 — Figure S1 Figure S2 Figure S3 [file CAM4-11-3364-s002.docx]

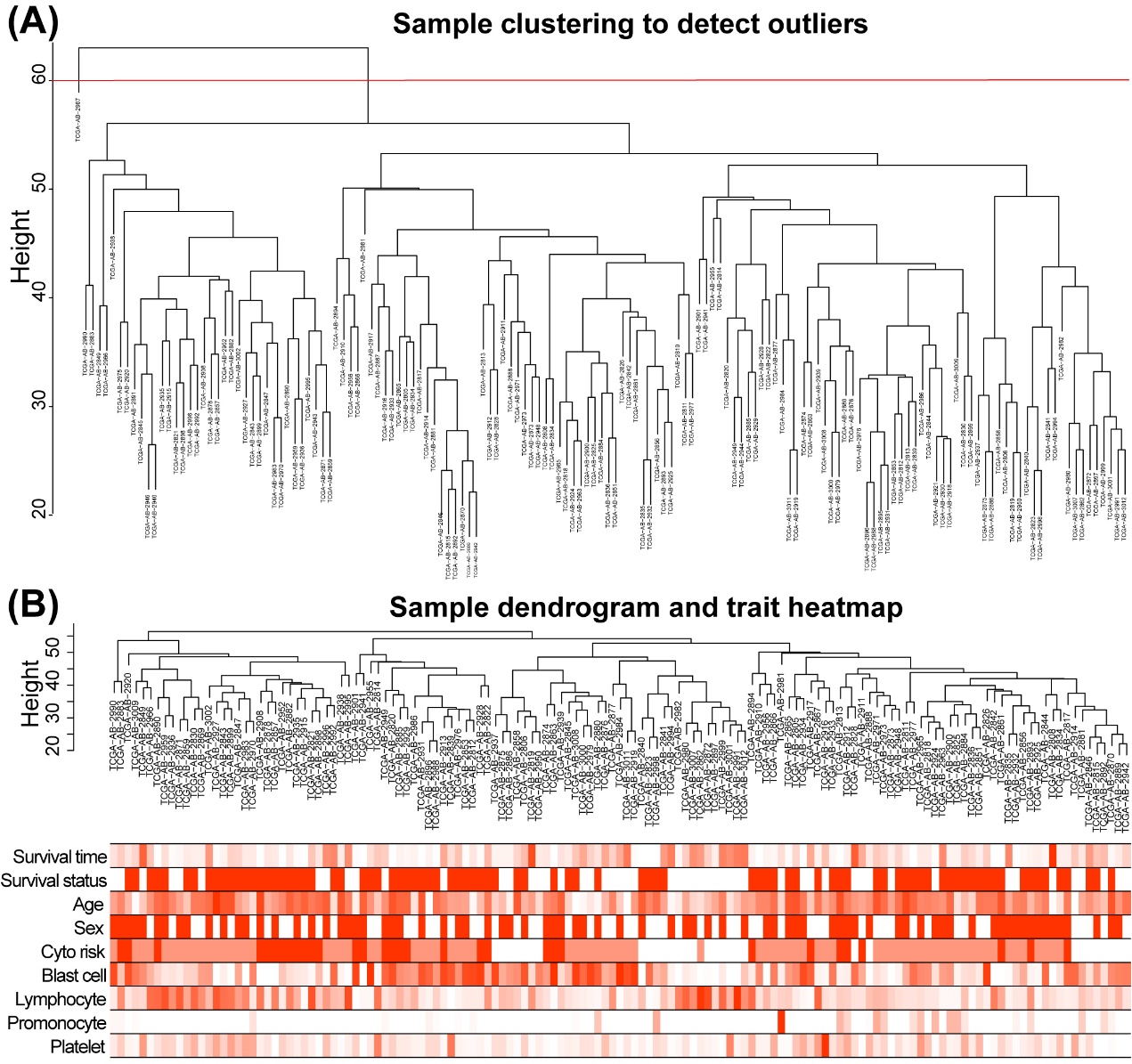


**Figure S1.** Hierarchical clustering of TCGA-AML samples. **(A)** Outliers were detected by hierarchical clustering. **(B)** Clinical traits were matched to the samples and displayed as a heatmap


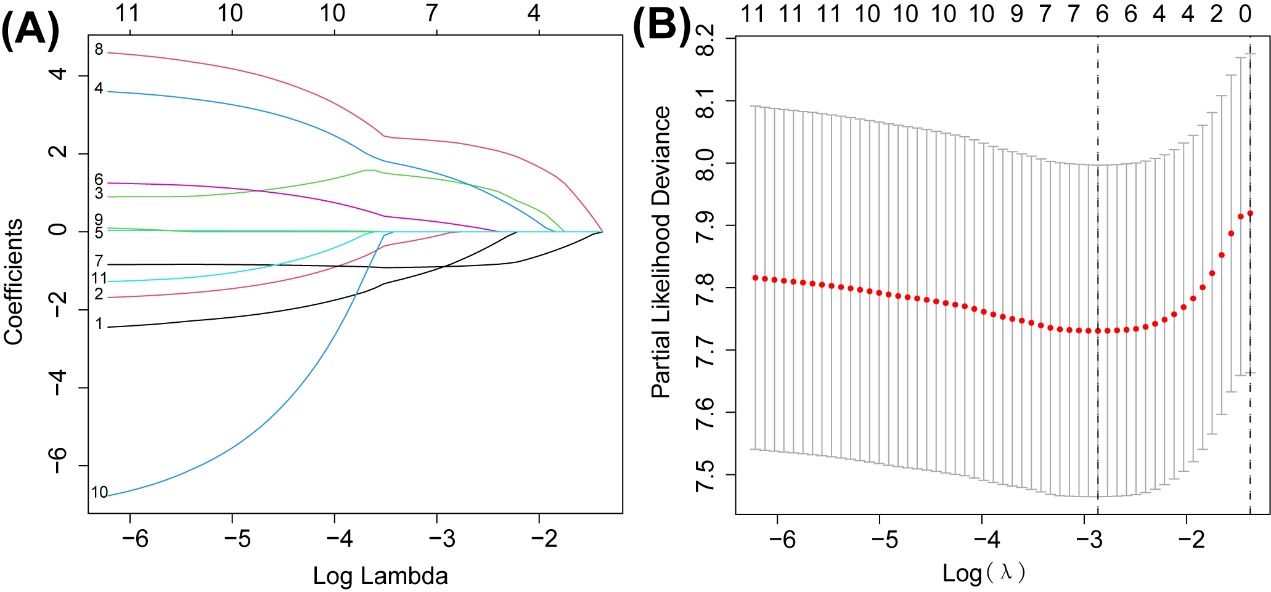


**Figure S2.** LASSO penalized regression. **(A)** LASSO model coefficient profiles of 11 immune-related genes. **(B)** Tuning parameter selection was performed through 10-fold cross-validation with minimum standards in the LASSO regression model

**
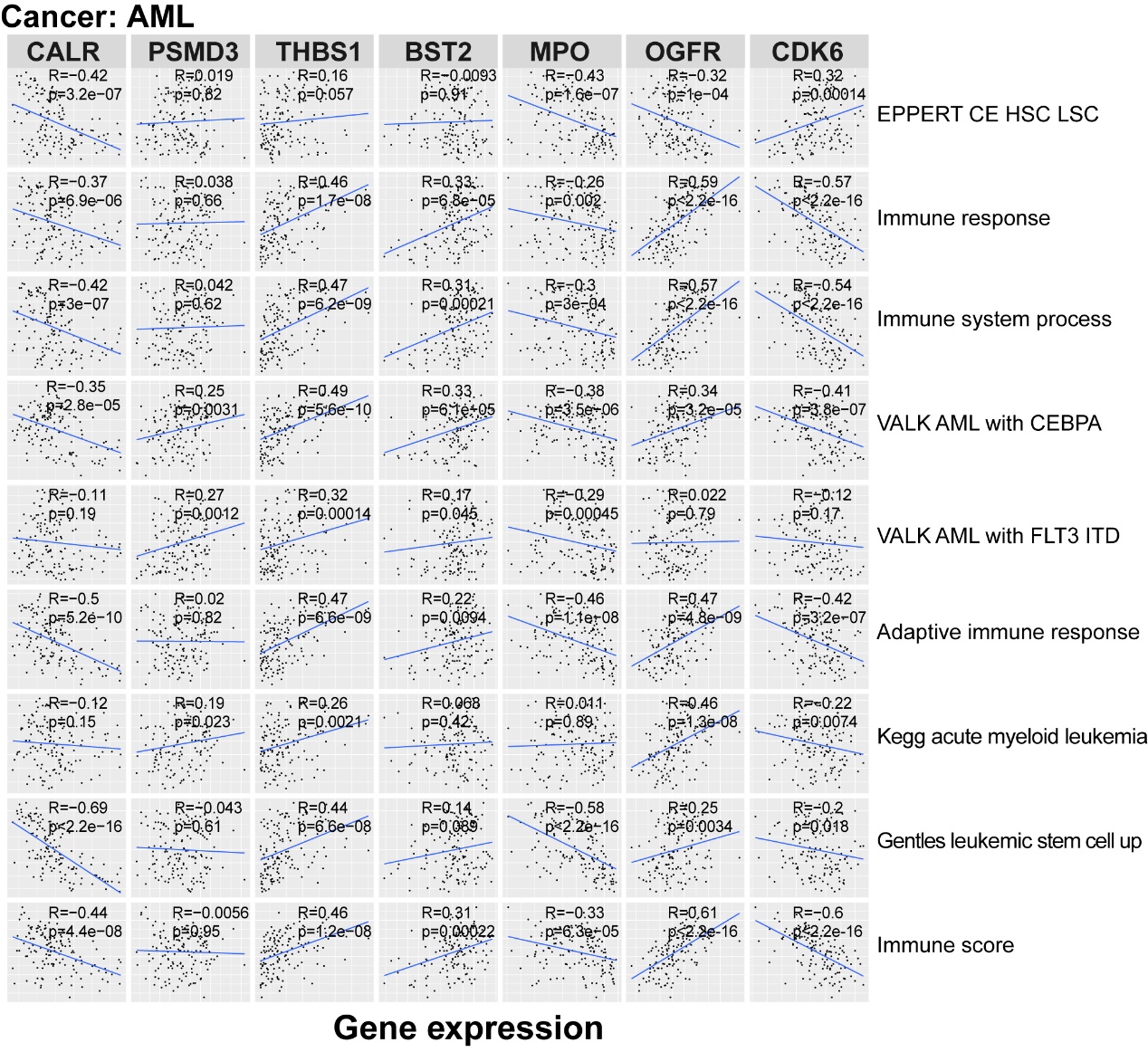
Figure S3.** The correlation between pathway activity scores and gene expression values
